# PharmDock: a pharmacophore-based docking program

**DOI:** 10.1186/1758-2946-6-14

**Published:** 2014-04-16

**Authors:** Bingjie Hu, Markus A Lill

**Affiliations:** 1 Department of Medicinal Chemistry and Molecular Pharmacology, College of Pharmacy, Purdue University, 575 Stadium Mall Drive, West Lafayette, IN 47906, USA

**Keywords:** Protein pharmacophores, Docking, Scoring, Biased docking, Constraint docking, Confined docking, GUI, PyMOL

## Abstract

**Background:**

Protein-based pharmacophore models are enriched with the information of potential interactions between ligands and the protein target. We have shown in a previous study that protein-based pharmacophore models can be applied for ligand pose prediction and pose ranking. In this publication, we present a new pharmacophore-based docking program PharmDock that combines pose sampling and ranking based on optimized protein-based pharmacophore models with local optimization using an empirical scoring function.

**Results:**

Tests of PharmDock on ligand pose prediction, binding affinity estimation, compound ranking and virtual screening yielded comparable or better performance to existing and widely used docking programs. The docking program comes with an easy-to-use GUI within PyMOL. Two features have been incorporated in the program suite that allow for user-defined guidance of the docking process based on previous experimental data. Docking with those features demonstrated superior performance compared to unbiased docking.

**Conclusion:**

A protein pharmacophore-based docking program, PharmDock, has been made available with a PyMOL plugin. PharmDock and the PyMOL plugin are freely available from http://people.pharmacy.purdue.edu/~mlill/software/pharmdock.

## Background

Pharmacophore models aim to comprise the features of ligand-protein interactions that are most crucial for binding and biological activity. These models are used for virtual screening (VS) to identify potential new actives or for generating ligand alignments for subsequent QSAR simulations. Pharmacophore models are typically derived from structural features common to biologically active ligands that are hypothesized to be important for biological activity [1-5]. Such ligand-based pharmacophore models are dependent on the chemical features present in the known actives. Physicochemical features that are absent in the particular set of actives, but are important for the binding of structurally different ligands, will likely be neglected in the pharmacophore model. Alternatively, the binding site of the target protein can be used to generate a protein-based pharmacophore model without the inclusion of ligand information. These protein-based pharmacophore models are advantageous because a priori knowledge of active ligands is not required and the models are not biased by the chemical space of previously identified actives. Several approaches [6-9] have been developed to derive protein-based pharmacophore models from ligand-free proteins and apply the pharmacophore models in virtual screening.

Protein-based pharmacophore models are, by definition, enriched with the information of potential interactions between ligands and the protein target. Consequently, a direct application of the protein-based pharmacophore models is to use them for ligand pose prediction and pose ranking. In a recent study, we have explored the potential of protein-based pharmacophore models in ligand pose prediction and ranking [10]. We carefully optimized the pharmacophore-generation process to reproduce native contacts for a large number of experimentally-determined protein-ligand complexes. We then developed a fast pharmacophore-based matching and scoring scheme and tested it on the PDBbind [11] core set. When the native ligand conformations, i.e. the ligand conformations from the crystal structures, were used as input, our pharmacophore-based scheme was able to identify native-like poses (with RMSD to the X-ray pose ≤ 2 Å) within the top-100 ranked poses for 94% of the tested entries. When the low-energy conformations generated by OpenEye Omega [12-14] were used as input, we were still able to maintain a success rate of 71% for predicting native-like binding pose within the top-100 ranked poses. These results were comparable in quality to several widely used docking programs.

Inspired by the promising results in ligand pose prediction and pose ranking using protein-based pharmacophore models, we extended the pharmacophore-based matching and scoring scheme into a docking program, named PharmDock (*Pharm*acophore-based *Dock*ing). The docking program further optimizes the top ranked binding poses predicted from the pharmacophore-based scheme and re-scores the optimized binding poses with a widely used empirical scoring function. We report here PharmDock’s performance in binding pose prediction and free energy of binding estimation tested on the PDBbind core set [11,15], as well as its performance in virtual screening on 29 targets from the dictionary of useful decoys (DUD) dataset [16]. We also present an open-source graphical user interface (GUI) adapted to PyMOL [17,18] that we have developed for PharmDock for ease use of the docking software by the scientific community. In addition, we developed two new features within the PyMOL GUI allowing the users to guide the docking process towards specific residues identified from previous experimental data.

## Implementation

### The docking program PharmDock

**Figure 1:**
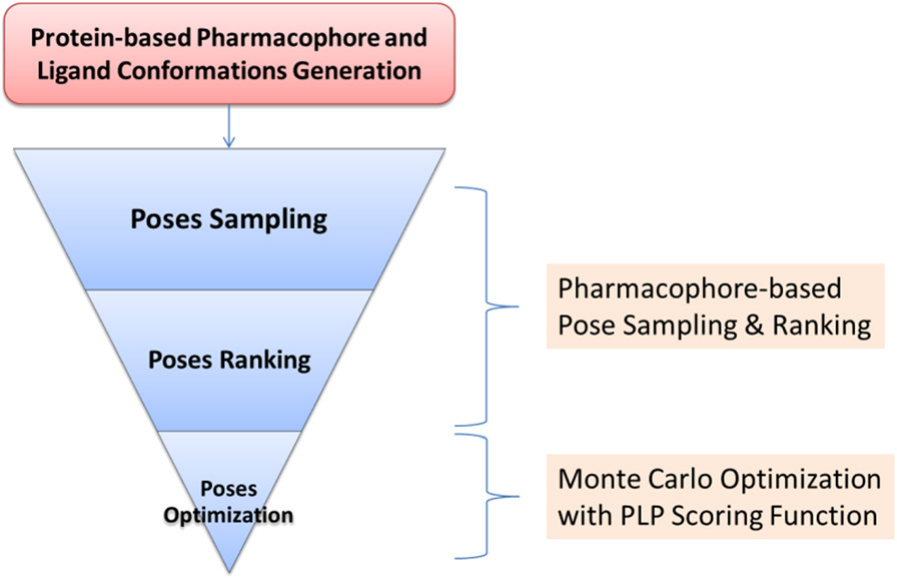
**Overview of PharmDock.** The inverse triangle shows that with progress in the overall docking process, the number of feasible binding poses of a ligand will be reduced. The overall docking process contains the following subsequent steps: Poses sampling: PharmDock samples binding poses by enumerating all possible multiple-points matches between the pre-generated ligand and protein-based pharmacophores and subsequent alignment of common features. Poses ranking: The sampled binding poses are then ranked using a simple pharmacophore-based scoring function (equation 1) to select top ranked poses for subsequent optimization. Both Poses Sampling and Poses Ranking are based on the representation of potential interactions as potential pharmacophores. Poses optimization: The top ranked binding poses will be locally optimized within the protein binding site to generate the final prediction of ligand binding pose and binding score.

An overview of PharmDock is shown in Figure 1. It samples the ligand binding poses by enumerating all possible multiple-points matches between pharmacophores of an ensemble of pre-generated ligand conformations and protein-based pharmacophores. The sampled binding poses are then ranked using a simple pharmacophore-based scoring function. A set of top ranked binding poses will be locally optimized within the protein binding site to obtain the final ligand binding pose and binding score. The first two parts of PharmDock are based on a pharmacophore or functional group representation of ligand and protein whereas the last step of pose optimization is atom-based. The details of pharmacophore generation, ligand poses sampling and ranking, final poses optimization will be described below.

#### Generation of protein-based pharmacophores

Protein-based pharmacophores refer to the potential interaction sites for the ligand to favorably interact with the protein atoms in the binding site. They can be viewed as the negative or complementary image of the topology and physicochemical properties of the protein binding site. Four types of protein-based pharmacophores are defined in our program: hydrogen-bond donor/acceptor, hydrophobic, aromatic and ionic pharmacophores. In addition, the exclusive volume of the protein is also represented by the so-called forbidden pharmacophores, representing the binding site residues that would sterically overlap with ligand atoms placed at this respective position.

The detailed method in generating protein-based pharmacophores has been described in our previous paper [10]. Briefly, the binding site of the protein is defined based on its known active ligand with a minimum of 3 Å to any ligand heavy atom. A 3D grid with 0.4 Å spacing between grid points was placed in the binding site for each protein structure. The interaction potentials (hydrogen-bond donor/acceptor, hydrophobic, aromatic and ionic) between the protein atoms and probes representing hypothetical ligand atoms were computed on each grid point. The interaction potentials for hydrogen-bonding and hydrophobic probes placed at the grid points were computed using a continuous form of the ChemScore [19,20] scoring function. The aromatic and ionic interactions were calculated using a functional form similar to ChemScore. The detailed equations can be found in the previous publication [10]. The pharmacophores were generated using the computed interaction energies with the probes on the 3D grid points. The hydrophobic pharmacophores were computed by a k-means clustering over all grid points with favorable hydrophobic scores. For each cluster, the hydrophobic pharmacophore element was then defined as the energy-weighted geometric center of all grid points of a particular cluster. The number of clusters, *k,* was adjusted until the minimum distance between a cluster center *i* and any other cluster center was on average smaller than a certain distance cutoff. K-means clustering to generate hydrogen-bond, aromatic and ionic pharmacophores was performed over the grid points associated with the same nearest functional group. For example, in generating a hydrogen-bond donor pharmacophore, the program iterates through all protein acceptors, and groups the grid points closest to the same acceptor into one patch. K-means clustering was then performed within this patch. In our previous study [10], we have investigated the influence of clustering distance cutoff of each pharmacophore type on the ligand pose sampling accuracy and efficiency. We found that pharmacophore models comprised by only hydrophobic and hydrogen bond elements, which are generated using a distance cutoff of 1.5 Å and 2.0 Å respectively, provide the best compromise between pose sampling accuracy and efficiency. These values will be used for the pharmacophore-based pose sampling process described below. For the pose-ranking process, a more detailed pharmacophore model using a 1 Å cluster distance cutoff for all pharmacophore types was adopted. The rationale is that the densest pharmacophore model provides the best description of the potential protein-ligand interactions and consequently should provide the largest amount of information for scoring.

#### Generation of ligand conformation and pharmacophores

PharmDock uses the low-energy conformers for a ligand generated by Openeye Omega [12-14] as docking input. For each ligand, a maximum of 100 conformations are generated with the calculated internal energy no more than 15 kcal/mol above the energy of the ligand conformation with the lowest internal energy. Duplicate conformers are removed using a 0.2 Å root-mean-square deviation (RMSD) cutoff for ligands with zero to three rotatable bonds, a 0.3 Å cutoff for ligands with four to six rotatable bonds, and a 0.4 Å cutoff for all ligands with more than six rotatable bonds. The *in-house* program *clusterconformer* is then used to generate the pharmacophore elements for each ligand conformation. Four types of pharmacophores are defined for each ligand: hydrogen-bond donor/acceptor, hydrophobic, aromatic and ionic pharmacophores. Hydrogen-bond pharmacophores are placed at the position of potential donor and acceptor groups of the ligand: Hydrogen-bond donors are polar hydrogen atoms bonded to oxygen, nitrogen and sulfur atoms, acceptors are oxygen, nitrogen and sulfur atoms with at least one lone pair. Ligand atoms (excluding hydrogen atoms) are defined to be hydrophobic if they were not hydrogen-bond donors or acceptors or directly bonded to a ligand’s donor or acceptor atoms. The hydrophobic atoms from each ligand conformation are clustered using hierarchical clustering with a minimum distance between cluster centers of 2.0 Å. Clustering is performed to reduce the number of hydrophobic ligand pharmacophores. This significantly reduces the cost of clique detection and consequently increases the efficiency of the docking process. Aromatic pharmacophores are defined as centers of aromatic rings. Ionic groups included functional groups that are formally charged positive or negative, e.g. protonated amines or deprotonated carboxylic acids, and are placed at the centroid of the functional group.

#### Pharmacophore-based pose sampling and ranking

The binding pose sampling and ranking process of PharmDock has been described and discussed in our previous publication [10]. To provide the best compromise between accuracy and efficiency, only hydrophobic and hydrogen bond pharmacophore elements were used for the pose sampling process. This can be substantiated by our observation that on average only one aromatic interaction and less than one ionic interaction per protein - ligand complex are present in the 190 protein - ligand complexes we examined [10]. This is in contrast to an average of four H-bond and ten hydrophobic interactions that were observed in the same dataset. A detailed evaluation of the pose sampling process can be found in our previous paper [10]. Briefly, the pose sampling is based on a modified Bron-Kerbosch clique detection algorithm [21,22] that enumerates all possible multi-points (> = 3) matches of ligand and protein-based pharmacophores. First, the length of the edge between each pair of ligand pharmacophores is determined. The edge length is also determined for each protein-based pharmacophore pair. All ligand pharmacophore edges that match the protein-based pharmacophore edges, based on the pharmacophore types (hydrogen bond donor/acceptor and hydrophobic) of their vertices and edge lengths, are identified. Throughout the matching process, a tolerance of 0.3 Å for the edge lengths is allowed. The matching process can be represented by a graph in which each node represents a matching ligand-protein pharmacophore pair. The clique detection algorithm then identifies all the completely connected subgraphs from this graph. The Kabsch algorithm [23] is then used to spatially align the ligand pharmacophore elements to the matching protein-based pharmacophores in each clique, thus placing the ligand into the protein binding site. To avoid steric clashes between ligand and protein atoms, the number of heavy atoms of the ligand that are located within 1.3 Å to any of the forbidden pharmacophores is counted for each ligand pose. If more than 10% of the ligand’s heavy atoms overlapped with forbidden pharmacophores, the pose is rejected.

The ligand poses sampled by PharmDock are initially scored and ranked using a simple geometric function based on the matching pharmacophore pairs formed by each ligand pharmacophore and its closest protein-based pharmacophore of the same type:

(1) $$\begin{array}{ll} S= & ‒0.7*\sum_{\mathrm{hbond}}f\left(r\right)‒0.4*\sum_{\mathrm{hphob}}f\left(r\right)‒0.6\\ & *\sum_{\mathrm{arom}}f\left(r\right)‒0.6*\sum_{\mathrm{ionic}}f\left(r\right) \end{array}$$

The weights for the different types of pharmacophores were optimized to achieve the best separation between native-like poses (≤2 Å RMSD to the X-ray binding pose) and decoy poses for 190 tested protein-ligand complex structures. A detailed description of the optimization procedure can be found in our previous publication [10]. f(r) is a distance-dependent function that measures the spatial separation of ligand and protein-based pharmacophores of a matching pharmacophore pair:

(2) $$f\left(r\right)=\left\{\begin{array}{cc} 1.0 & r\leq 0.5Å\\ 2*\left(1.0-r\right) & 0.5Å<r\leq 1.0Å\\ 0 & r>1.0Å \end{array}\right)$$

r is the distance between the ligand pharmacophore and its closest matching protein-based pharmacophore of the same type. It is noteworthy that equation 2 calculates the score of a pose based on all the ligand pharmacophores rather than only those involved in forming the matching cliques. Also, the detailed pharmacophore models with 1 Å clustering distance cutoff were used for the ranking process.

#### Local optimization of the ligand binding poses

The pharmacophore-based pose sampling and ranking scheme was deployed to efficiently filter out ligand binding poses that are unlikely to be the native binding pose. As shown in our previous study [10], this procedure was indeed very effective to enrich the ranking list of binding poses with native-like poses within the top-100 positions. To further optimize the predicted ligand binding poses and estimate the binding energy of the ligand to the target, we perform a local Metropolis Monte Carlo (MC) [24] optimization for the top-100 ranked poses with the Piecewise Linear Pairwise (PLP) scoring function [25].

PLP [25] is one of the earliest developed empirical scoring functions. It is a sum of pairwise interactions between protein and ligand atoms based on their interactions types. Only hydrogen bonding (H-bond) and steric interactions are considered:

(3) $$E_{\mathrm{total}}=E_{H-\mathrm{bond}}+E_{\mathrm{steric}}$$

A detailed description of the PLP function can be found in Gehlhaar *et al*.’s [25] original work. Briefly, each ligand and protein atom is categorized into four types: H-bond donor, H-bond acceptor, H-bond donor/acceptor, nonpolar. Each pair of ligand-protein atoms is then assigned with one and only one of the interaction types: H-bond or steric. Both *E*_*H* - *bond*_ and *E*_*steric*_ are computed using similar piecewise linear functions but with different parameters [25]. Despite the simplicity of PLP, it was shown to be one of the best scoring functions in identifying the true ligand binding poses among decoy poses and ranking the different ligands that bind to the same protein according to their binding affinities [26]. Therefore, we finally settled on PLP scoring function for our local optimization and final scoring of the ligand binding poses.

An individual MC optimization was performed for every of the top-100 binding poses generated by clique detection and ranked by the simple pharmacophore-based scoring function. Throughout MC refinement for a given ligand binding pose, a new ligand pose is generated from the previous pose by applying small perturbations on its position, orientation and torsion angles. The newly sampled pose is accepted based on several criteria: 1) If the RMSD of the current ligand pose compared to the starting pose is larger than 3.5 Å, the current pose is rejected and coordinates of the starting pose are reassigned to the ligand. 2) If the RMSD is smaller than 3.5 Å, the PLP score of the current pose (*Score*_*cur*_) is compared with the minimum PLP score of all previous poses (*Score*_*min*_). If *Score*_*cur*_ is smaller than *Score*_*min*_, the current step is accepted. Otherwise, the *Score*_*cur*_ is compared with the score of the last accepted step (*Score*_*pre*_) and the current pose is accepted based on the probability *P*:

(4) $$P=\exp\left(-\frac{\mathrm{Scor}e_{\mathrm{cur}}-\mathrm{Scor}e_{\mathrm{pre}}}{\mathrm{RT}}\right)$$

where *R* is the ideal gas constant (1.986 cal/mol-K) and *T* the temperature (300 K). If this factor is larger than a random number generated from a uniform distribution between 0 and 1, the current pose is accepted.

During the MC simulation, several strategies are implemented to avoid getting trapped in a local minimum or pose that has steric overlap with the protein. First, the size of maximum allowed translational, rotational and torsional changes at each MC step is adapted using the previously observed acceptance rate. If the acceptance rate over the last 100 MC steps is below 0.4, the size of maximum allowed changes is decreased, if the rate is above 0.6, the size is increased. Second, if the program rejects the pose from three consecutive MC steps, the ligand is set back to its original conformation. Third, different maximum step sizes for central and terminal torsions are determined based on the equation:

(5) $$\mathrm{ratio}=\left\{\begin{array}{c} \left(1.0+\cos\left(\pi \times \frac{N_{\mathrm{atoms}}}{N_{\max}}\right)\right)\times 0.5\times \left(1.0-\epsilon \right)+ \end{array}\right)\begin{array}{r} \epsilon ,\left|N_{\mathrm{atoms}}<N_{\max}\right)\\ \epsilon ,\left|N_{\mathrm{atoms}}\geq N_{\max}\right) \end{array}$$

Where ϵ is set to 0.15 and *N*_*max*_ is 8. *N*_*atoms*_ is the total number of heavy atoms attached to the smaller branch of this torsion. Equation 5 determines a scaling factor ‘*ratio*’ with which the maximum allowed torsional change is modified. For example, if the maximum torsional change is set to *max* = 60°, this value will be scaled by *ratio =* 0.5 + 0.5*ϵ* = 0.575 (yielding *max* = 34.5°) and *ratio* = 0.15 (*max* = 9°) for torsions with 4 and 8 heavy atoms attached to the smaller branch of the torsion, respectively.

#### Development of the PyMOL plugin

**Figure 2:**
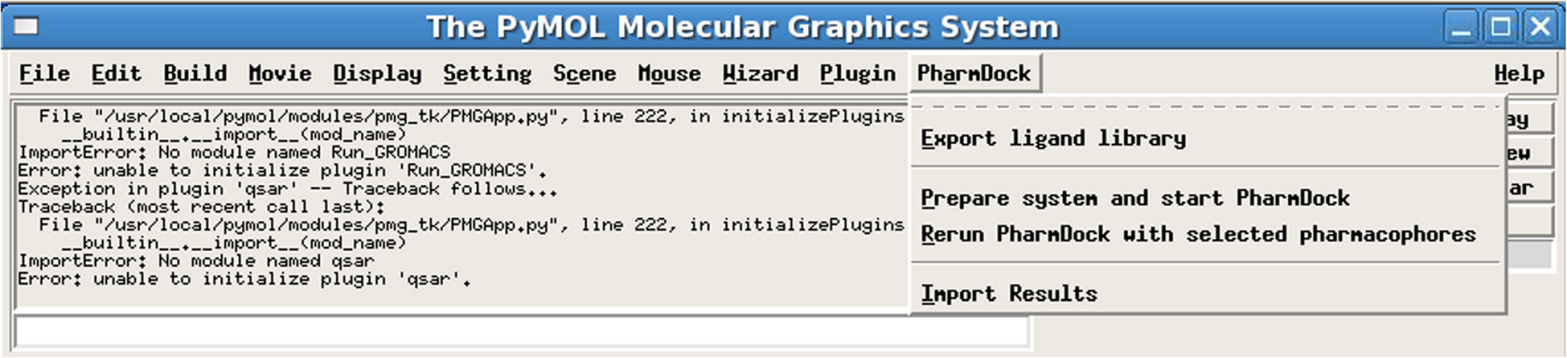
**Illustration of PharmDock main menu with different sub-processes.** Exporting ligand libraries, performing docking calculations, either unbiased or biased, and importing docking results into PyMOL for visual analysis were shown.

**Figure 3:**
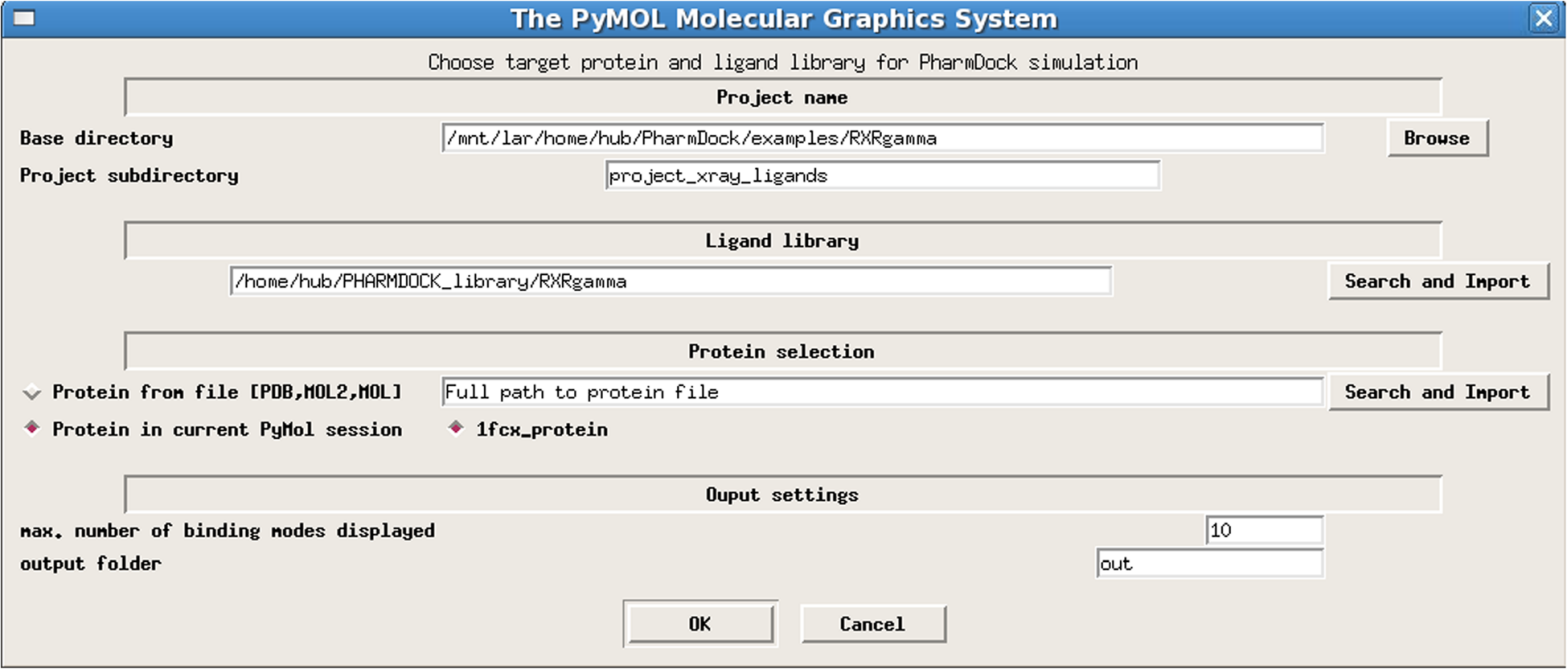
**Illustration of docking settings.** The dialog specifies the location where docking results are stored, the ligand library and protein file used for docking, the option for binding site definition, and options for output of docking results.

**Figure 4:**
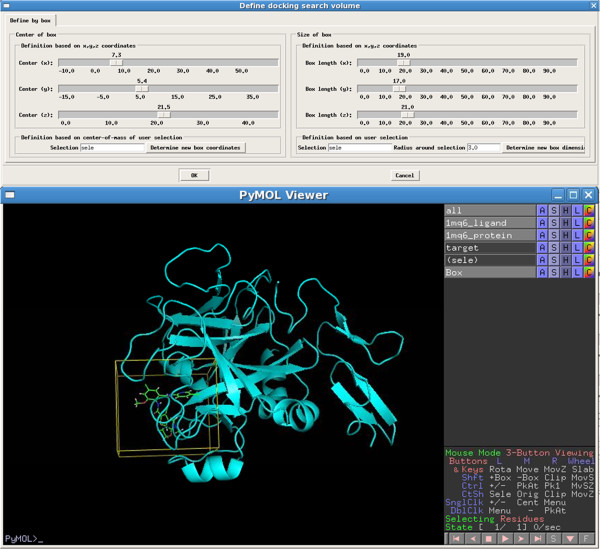
**Illustration for the definition of search volume for PharmDock.** The docking search volume can be defined by visually adjusting (top) the position and size of the box displayed in PyMOL (bottom).

For easy use of PharmDock, a GUI plugin of PyMOL was built using the Python programming language. The Python script is located in the startup folder of PyMOL to allow for automatic load and display of the submenu “PharmDock” (Figure 2) within the standard PyMOL menubar. The plugin features the generation of ligand libraries by exporting all ligand objects present in a PyMOL session. A lexicon of exported libraries is stored and each library of compounds can later be modified, combined with other libraries, and imported for docking to different target proteins. The submenu “prepare system and start PharmDock” directs the user to select the target protein, import the ligand library, define the protein binding site and settings for the output of docking results (Figure 3). Users can define the docking search volume by visually adjusting the position and size of the box displayed in PyMOL (Figure 4).

The docking calculations are performed in the background after submitting the job. The location of the docking results is stored in a monitoring file that allows the user to check the progress of the docking runs and re-import the docking results into PyMOL. All docking poses will be automatically displayed to the user with a separate dialog displaying the docking solutions and associated docking scores.

**Figure 5:**
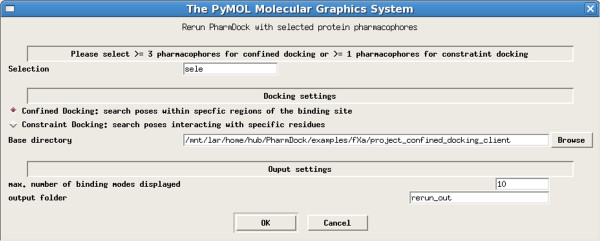
**Illustration of PharmDock’s settings for confined or constraint docking.** Pharmacophores can be visually selected with the PyMOL GUI.

In the default setting, PharmDock searches the ligand binding poses using all protein-based pharmacophores independent from information about known active ligands. In this setting, docking results are not biased towards the chemical space of previously identified active ligands. However, it is possible that researchers would like to use previously acquired empirical knowledge about the target and identify compounds that can form specific interactions with certain region or specific residues of the binding pocket. To allow for inclusion of such information, we provide the users with the option to perform docking with PharmDock focusing on a set of selected pharmacophore elements (Figure 5). The GUI will allow users to load protein-based pharmacophore files and select critical pharmacophores for protein-ligand binding. Two options are available to guide docking towards the selected pharmacophores: Confined docking, where the search volume will be confined to include only the selected pharmacophores; and constraint docking, where the generated docking poses must match at least one of the selected pharmacophores but the search volume is unmodified compared to the original unbiased docking. Confined docking is designed for the purpose of “confining” the docking poses within certain regions of the binding pocket and the confined region can be defined by using the known active ligands. Constraint docking is designed for identifying ligands or ligand poses that form interactions with specific residues within the binding site. Examples for the two options will be shown in the “Results and Discussion” section.

### Tests of PharmDock’s docking performance

#### Cognate docking

The “core set” of the PDBbind [11,15] database (version 2007) was used to test PharmDock for its performance on ligand binding pose prediction and binding energy estimation. The PDBbind “core set” provides 210 protein-ligand complexes non-redundantly sampled from 1,300 protein-ligand complexes [15]. It covers 70 different proteins, each of which contains three protein-ligand complexes with different binding affinities. All the protein-ligand complexes in the PDBbind core set were pre-processed with hydrogen atoms added and were therefore used directly without additional preparations. Due to various reasons, 20 protein-ligand complexes were excluded from the pose prediction and ranking study as described in our previous study [10].

The performance of PharmDock in ligand binding pose prediction was evaluated by its ability to reproduce the native-like pose for each protein-ligand complex in the PDBbind core set at the top-1 position. The top pose RMSD, i.e. the RMSD between the top-1 ranked pose to the native binding pose denoted as RMSD_top_, was calculated. The average RMSD_top_ over the studied protein-ligand complexes was reported to assess the overall sampling performance. In addition, the percentages of complexes that were successfully predicted with RMSD_top_ within 1 Å, 2 Å and 3 Å to the native conformation were also used to evaluate the overall performance of pose generation and ranking. The correlation between PharmDock’s predicted binding energies with the experimentally measured binding affinities was used to evaluate PharmDock’s performance in binding energy estimation. And finally, the success rate of PharmDock in ranking three ligands bound to the same protein according to their binding affinities was also calculated.

#### Virtual screening

The dictionary of useful decoys (DUD) [16] dataset was used to perform virtual screening (VS) studies. The DUD dataset contains 40 protein targets and a set of active and decoy ligands corresponding to each target. In the current version of PharmDock, the parameters of ions and cofactors were not included. Therefore, the four metalloenzymes, two folate enzymes and five other enzymes (aldose reductase, enoyl ACP reductase, glycogen phosphorylase *β*, purine nucleoside phosphorylase and S-adenosyl-homocysteine hydrolase) were excluded in our VS experiment. For each protein structure in DUD, the side-chain conformations of ASN, GLN and HIS, and tautomers and protonation states of HIS were adjusted using the Reduce program [27]. The hydrogens were added to the protein using the tleap module of Amber 10 [28]. The protein-based pharmacophores and ligand conformations and pharmacophores were generated for each target following the methods described in “Generation of Protein-based Pharmacophores” and “Generation of Ligand Conformation and Pharmacophores”.

To analyze the VS results, the ligands for each protein system were ranked based on their predicted binding energies. The Receiver Operating Characteristic (ROC) curve displaying the fraction of ranked actives (true positive rate) at a given fraction of ranked decoys (false positive rate) was plotted for each VS run. The area-under-the-curve (AUC) was calculated for each ROC curve and used to assess the overall enrichment quality.

## Results and discussion

### Prediction of binding poses

**Figure 6:**
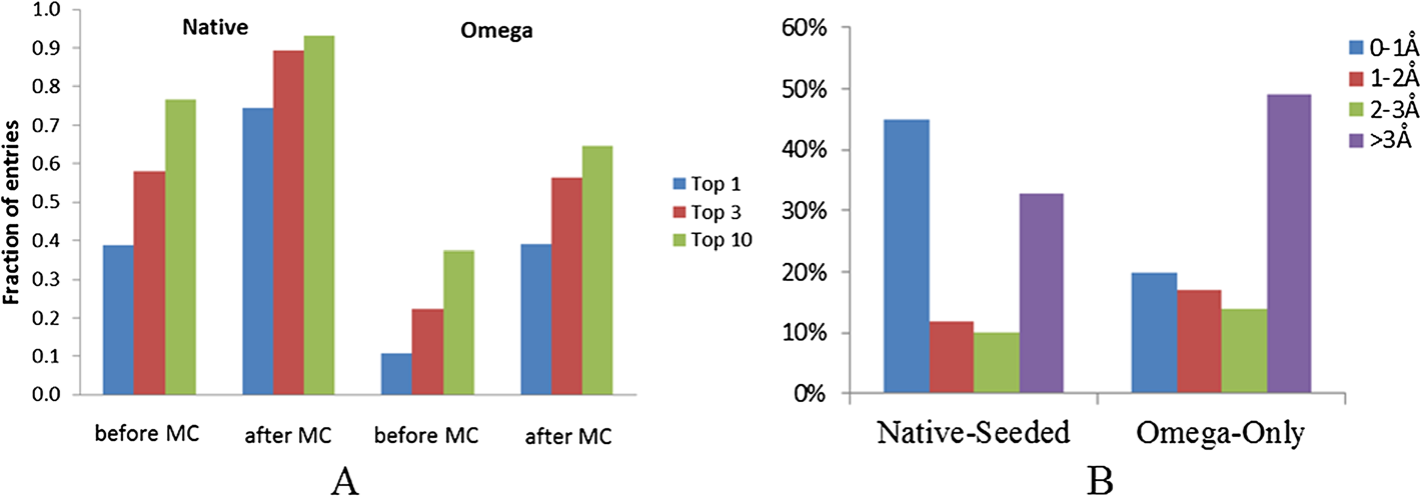
**Prediction of binding poses by PharmDock. (A)** Comparison between the docking performance of PharmDock with and without Monte Carlo (MC) optimization. The fraction of protein-ligand complexes that have at least one predicted pose within 2.0 Å RMSD to the native binding pose is displayed on the y-axis. The first two columns are the docking results using native conformer as the docking input. The last two columns are using the Omega-generated low-energy conformers as docking input. **(B)** Distribution of the RMSD_top_. Native-Seeded: Docking was performed with the native ligand conformation seeded within the Omega-generated low energy conformations as input. Omega-Only: Docking was performed with only the Omega-generated low energy conformations as input.

To evaluate PharmDock’s performance in predicting ligand binding poses in close agreement with the X-ray poses, we performed cognate docking studies on the PDBbind [11,15] core set. In the default setting, PharmDock uses the ligand conformations generated by OpenEye Omega [12-14] as docking input. Our previous study [10] and many other docking studies [29,30] have shown that the input ligand conformations can have a significant influence on the prediction of the binding poses. To assess the influence of the input ligand conformations on PharmDock, we performed two docking runs for each protein-ligand complex: one with the native conformation seeded within the low energy conformations of Omega (Native-Seeded) and one with only the low energy conformations (Omega-Only). In our previous study, we have presented pose prediction and ranking results using pharmacophore models without any local optimization of the binding poses or use of an atomistic scoring function. To demonstrate that the optimization process is effective in improving the docking power, we compared the pose prediction and ranking results with and without the MC optimization. When the native conformer was used as docking input, the fraction of entries that have correctly predicted poses (RMSD ≤ 2 Å to the native binding pose) at the top-1 position was nearly doubled using MC optimization compared to pharmacophore matching only (Figure 6A). The fraction of entries that have correctly predicted poses within the top-10 ranked poses was 92%. When using Omega generated low-energy conformers as docking input the fraction of entries with correctly predicted poses at the top-1 position increased significantly from 10.9% without MC optimization to 39.1% with MC optimization. A nearly three-fold increase was also observed for the top-3 ranked poses. In summary, the MC optimization combined with PLP scoring function significantly improves the ability of PharmDock to identify the native binding pose of a ligand.

A detailed analysis of the distribution of the RMSD values between the top-1 ranked binding pose and the X-ray binding pose (RMSD_top_) for PharmDock with MC optimization is shown in Figure 6B. When the native ligand conformations were provided together with the low-energy conformations, PharmDock was able to predict binding poses with RMSD_top_ ≤ 1 Å for 45% of the tested protein-ligand complexes. When the native ligand conformations were excluded from the docking inputs, this number dropped to 20%. Obviously, the starting ligand conformations are critical for obtaining the correct docking solutions in PharmDock. As demonstrated in our previous study [10], Omega was not always able to sample ligand conformations within 1 Å RMSD to the native ligand conformation. Under our current setting (see “Generation of Ligand Conformation and Pharmacophore” in Methods section), 67% ligands had at least one conformer generated within 1 Å RMSD to its native conformation. This lack of generating native ligand conformations with Omega for a significant fraction of the docked ligands was the major reason for the dramatic drop in the success rate after excluding the native conformation from the docking inputs.

We compared the performance of PharmDock in predicting the ligand binding poses with the results of seven widely used docking programs evaluated by Plewczynski *et al.* on the PDBbind refined set [31]. The average RMSD_top_ (avg RMSD_top_) and the fraction of protein-ligand complexes that have RMSD_top_ ≤ 2 Å (%entries ≤ 2 Å) are shown in Table 1. When the native conformations were provided together with the low-energy conformations in docking, PharmDock was able to predict a correct binding pose (RMSD ≤ 2 Å) for 56% of the complexes with an average RMSD_top_ of 2.9 Å. This result was among the best of the compared docking programs. When the native conformation was excluded from the docking input of PharmDock, the fraction of protein-ligand complexes with correctly predicted binding poses was significantly reduced (37%). But taking the average RMSD_top_ into consideration, the performances of PharmDock were still comparable to that of Glide and AutoDock, and better than LigandFit and FlexX.

**Table 1:** **Comparison of PharmDock with docking programs evaluated by Plewczynski *et al*.**

	**Native conformation as docking input**^**1**^		**Omega conformations as docking input**^**2**^	
	**avg RMSD**_**top **_**(Å)**	**%entries ≤ 2 Å**	**avg RMSD**_**top **_**(Å)**	**%entries ≤ 2 Å**
Surflex	3.2	52%	3.1	51%
GOLD	2.8	55%	2.4	63%
eHiTs	N/A	N/A	2.6	58%
Glide SP	3.3	52%	3.7	43%
AutoDock	2.3	56%	4.0	41%
LigandFit	3.2	48%	4.4	33%
FlexX	4.2	41%	4.3	37%
**PharmDock**	**2.9**	**56%**	**3.9**	**37%**

Avg RMSD_top_: RMSD_top_ averaged over all the tested complexes. %entries ≤ 2 Å: the fraction of complexes with RMSD_top_ ≤ 2 Å. The results for the seven docking programs in comparison were extracted from Plewczynski *et al*.’s study [31]. ^1^In Plewczynski *et al*.’s study, only the native ligand conformation is provided. In PharmDock simulations, the native ligand conformation is seeded within the Omega-generated low energy conformations, because PharmDock does not generate ligand conformations on-the-fly during pose sampling. Conformations are further modified during pose optimization. All other docking programs take the input ligand conformation and re-generate multiple ligand conformations during the search process. ^2^Plewczynski *et al*. generated ten low-energy conformers per ligand using Omega for their study.

We recognized that the dataset used in our study was not exactly the same as used by Plewczynski *et al.*. However the “core set” we used was a subset non-redundantly sampled from the “refined set” used by Plewczynski *et al.*. To further substantiate our comparison, we compared PharmDock with four docking programs (Glide, GOLD, LigandFit and Surflex) evaluated by Li *et al.* on the PDBbind core set [29]. As shown in Table 2, when the native ligand conformers were provided in the docking inputs, PharmDock was still among the best of the studied docking programs. When only the low energy conformers were provided, PharmDock performed comparably to Surflex and LigandFit. This is consistent with the previous comparison against Plewczynski *et al.*’s study.

**Table 2:** **Comparison of PharmDock with docking programs evaluated by Li *et al*.**

	**Native conformation as docking input**^**1**^	**Low energy conformations as docking input**^**2**^
Glide XP	65%	48%
GOLD/GoldScore	58%	45%
GOLD/ChemScore	58%	43%
LigandFit	54%	36%
Surflex	51%	41%
**PharmDock**	56%	37%

Fraction of protein-ligand complexes with RMSD_top_ ≤ 2 Å for PharmDock in comparison with the four docking programs evaluated by Li *et al*. [29]. ^1^Similar as in Table 1, the result using PharmDock is shown for omega-generated conformations seeded with native conformation as input, whereas the results extracted from Li *et al.’s* study only take the native conformation as docking input. ^2^Li *et al.* used “CONFORT” in SYBYL to generate one low energy conformation per ligand for the study.

### Prediction of binding affinities

**Figure 7:**
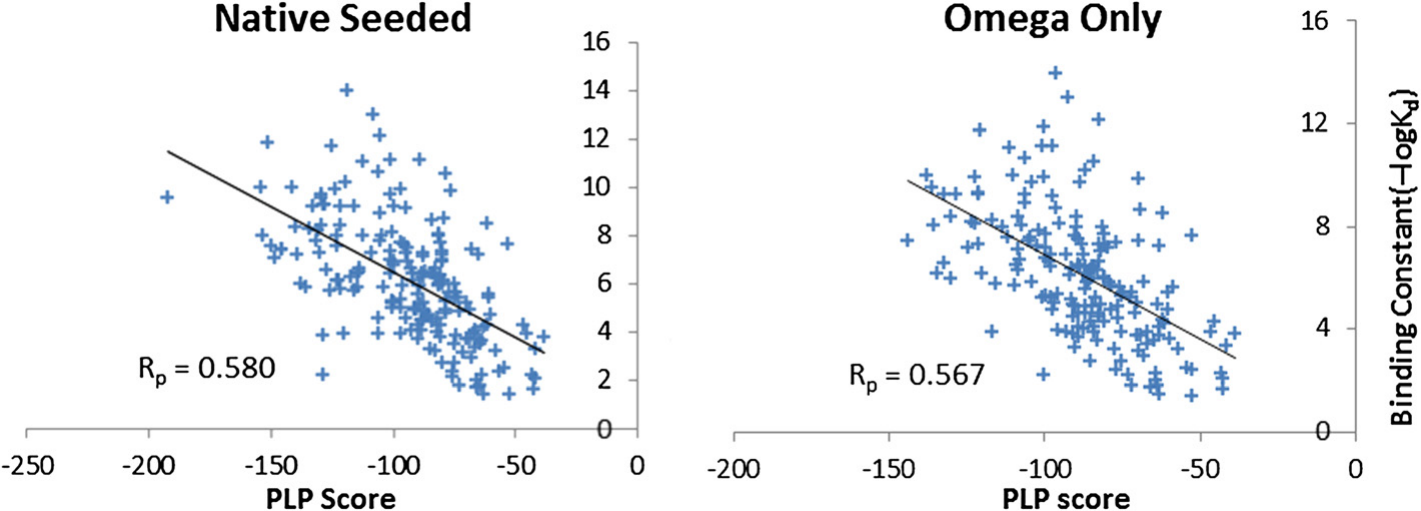
**Correlations between the experimentally measured binding constants of the protein-ligand complexes and the PharmDock-predicted binding scores.** The experimentally measured binding constants were shown in –log K_d_ units. **Left**. Correlations between the binding constants and the predicted binding scores when native conformers were provided together with low-energy conformers as input for docking. **Right**. Correlations between the binding constants and the predicted binding scores when using only low-energy conformers as docking input.

Another important evaluation of the docking program is how well the predicted binding energies correlate with the experimentally measured binding affinities. Experimentally measured binding affinities are available for all protein-ligand complexes provided in the PDBbind database. Figure 7 shows the correlations between the experimentally measured binding constants (in –log K_d_ units) and the predicted binding scores by PharmDock for all tested protein-ligand complexes. The Pearson correlation coefficients (*R*_*p*_) were 0.580 and 0.567 for the docking calculations based on native-seeded and omega-only input conformations, respectively. Previously, Cheng *et al. *[26] have performed a comparative study on 16 widely-used scoring functions using the PDBbind core set. They used these scoring functions to estimate the binding affinities of the protein-ligand complex structures as obtained from the X-ray experiments; thus, no pose sampling was performed removing the uncertainty typically generated in this step of standard docking protocols. The correlations between the predicted binding scores of individual scoring function and the experimentally measured binding affinities range from 0.644 to 0.216. The top three best correlations were obtained by X-score with *R*_*p*_ of 0.644, DrugScore with *R*_*p*_ of 0.569 and ChemScore with *R*_*p*_ of 0.555. Using PLP scoring function on the X-ray protein-ligand complexes an *R*_*p*_ of 0.545 was obtained. It is worth noting that the X-ray protein-ligand structures were used in Cheng *et al.’*s study whereas the top-1 ranked poses from PharmDock were used in our study for computing the binding scores. Despite the additional uncertainty in generating native poses in our study, a higher *R*_*p*_ (in both native-seeded and omega-only cases) was obtained compared to the study from Cheng *et al*.’s using the same PLP scoring function. One other significant difference between using PharmDock generated ligand poses and the crystal ligand poses is that in our study PharmDock MC optimization were performed to locally optimize the binding poses according to the PLP scoring function. Therefore, our results suggest that an *in situ* optimization with the final scoring function may be beneficial for reducing possible steric clashes in the original crystal structure and optimizing the beneficiary protein-ligand contacts.

The PDBbind “core set” contains three protein-ligand complexes for each protein target. We studied if PharmDock is able to correctly rank the three different ligands bound to the same protein according to their binding affinities. There are 70 different proteins in the original PDBbind core set. After removal of the 20 complexes, as described before, 55 proteins remained that still had three different complexes available for our ranking study. For 42% of these 55 protein targets in both native-seeded and omega-only cases PharmDock was able to correctly rank the three ligands according to their binding affinities. According to the results reported by Cheng *et al*., when the original crystal complex structures are used for estimating the binding energies, the highest success rate of ranking the ligands for the same protein target was 58.5%. Our 42% success rate ranks at the 9^th^ position compared to the 16 scoring functions tested by Cheng *et al*.. However, we need to mention again, that the top ranked docking poses rather than the X-ray conformations were used in our case, where the former is more complicated than the latter approach, as additional uncertainties are introduced throughout the pose sampling stage. Therefore a firm conclusion cannot be drawn directly from this comparison. However, combined with the comparable high correlation coefficients between the PharmDock predicted binding energies and the experimentally measured binding affinities, this result suggests that a good prediction in the overall correlation does not guarantee a good ranking power for the ligands bound with the same protein. A scoring function specifically developed for ranking the ligands bound to the same protein might be necessary for improving the performance of PharmDock in such studies.

### Virtual screening experiment

**Figure 8:**
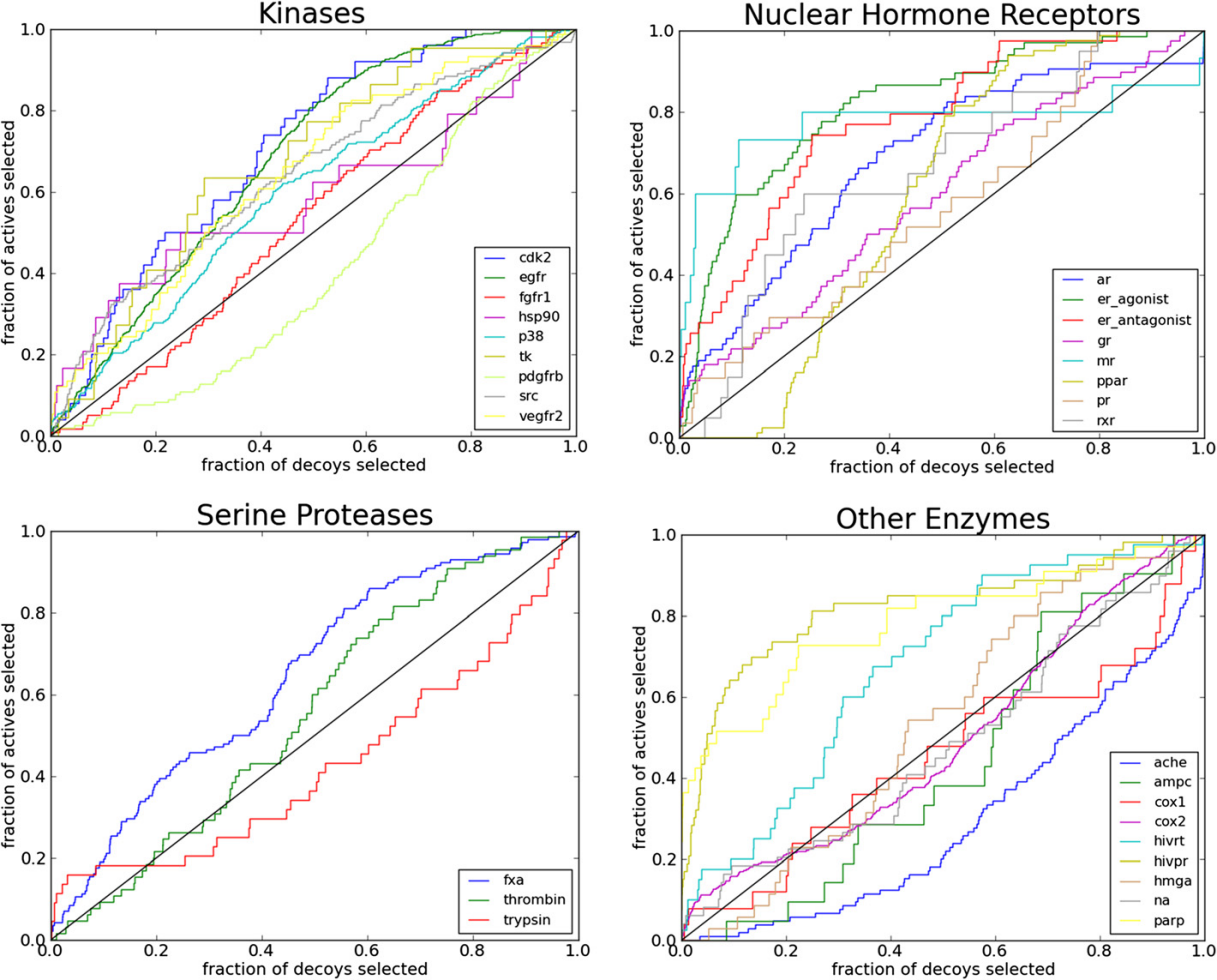
**ROC plots for 29 tested targets from the DUD dataset.** Diagonal line on each figure indicates the random performance. Enzyme abbreviations: AChE, acetylcholinesterase; AmpC, AmpC β-lactamase; AR, androgen receptor; CDK2, cyclindependent kinase 2; COX-1, cyclooxygenase-1; COX-2, cyclooxygenase-2; DHFR, dihydrofolate reductase; EGFr, epidermal growth factor receptor; ER, estrogen receptor; FGFr1, fibroblast growth factor receptor kinase; FXa, factor Xa; GR, glucocorticoid receptor; HIVPR, HIV protease; HIVRT, HIV reverse transcriptase; HMGR, hydroxymethylglutaryl-CoA reductase; HSP90, human heat shock protein 90; MR, mineralocorticoid receptor; NA, neuraminidase; P38 MAP, P38 mitogen activated protein; PARP, poly(ADP-ribose) polymerase; PDGFrb, platelet derived growth factor receptor kinase; PPARg, peroxisome proliferator activated receptor γ; PR, progesterone receptor; RXRa, retinoic X receptor α; SRC, tyrosine kinase SRC; TK, thymidine kinase; VEGFr2, vascular endothelial growth factor receptor.

**Figure 9:**
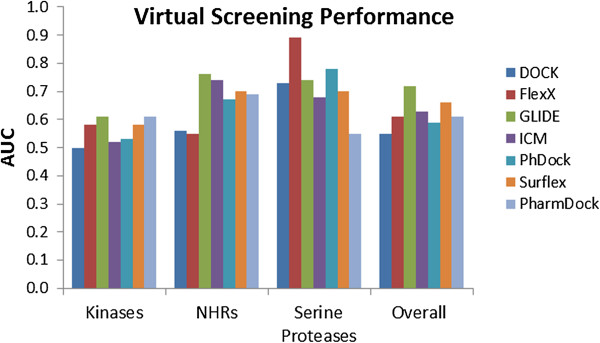
**Virtual screening performance of PharmDock in comparison with six other docking programs evaluated by Cross *et al*.**

The performance of PharmDock in retrieving active compounds from a virtual compound library was evaluated against 29 targets from the DUD data set. For each target, the “own decoy” set was used, which includes only decoys with physical properties similar to the native ligands. The overall virtual screening performance was evaluated by calculating the area-under-the-curve (AUC) value of the ROC curve plotted for each target system (Figure 8). In general, PharmDock provides an AUC above random for 22 out of the 29 tested targets. The average AUC among all the tested targets is 0.61. This value is comparable to those reported by Cross *et al*. [32] on the virtual screening performance of six docking programs (Figure 9). When breaking down our VS results into different protein families, PharmDock provides an average AUC of 0.61, 0.69 and 0.55 for kinases, nuclear hormone receptors (NHRs) and serine proteases respectively. PharmDock’s performance on kinases and NHRs are among the best in comparison with the other docking programs. Serine proteases turned out to be a difficult system for PharmDock. One possible reason for the rather weak performance of PharmDock on the serine proteases could be the neglect of solvation effects in the scoring function. Recent studies [33] on serine proteases suggest that the inclusion of explicit solvent effects is necessary to explain the structure-activity relationships of serine protease inhibitors. Therefore, a more sophisticated scoring function might be required to improve the performance of PharmDock on serine protease targets.

### Confined and constraint docking with PharmDock

To guarantee easy use of PharmDock, a PyMOL GUI was developed as described in the “Implementation” section. Besides the basic docking settings, the plugin also provides the user with the option to restrict docking to selections of the protein-based pharmacophores in order to concentrate the docking search volume to certain regions of the binding site. We termed this docking protocol as “confined docking”. Alternatively, the user can choose to utilize the “constraint docking” option, where at least one ligand pharmacophore of an accepted docking pose has to match with the user-selected pharmacophore elements. This option allows to enforce certain interactions that are critical for ligand binding to the target protein of interest. Typically, all pharmacophore elements of a property such as hydrogen-bonding are treated equally in the scoring function. The physicochemical environment of the hydrogen-bonding group or desolvation effects, however, can significantly alter the strength of a hydrogen bond [34]. Pharmacophores derived from an analysis of existing protein–ligand structures or previously identified active compounds can be used to differentiate between important and weak hydrogen bonds. The pharmacophores can be incorporated as constraints into docking, thus binding modes that do not match any of the selected pharmacophores will be discarded. To demonstrate how these two novel docking features can benefit the docking performance, examples of using the confined and constraint docking are shown in this section.

**Figure 10:**
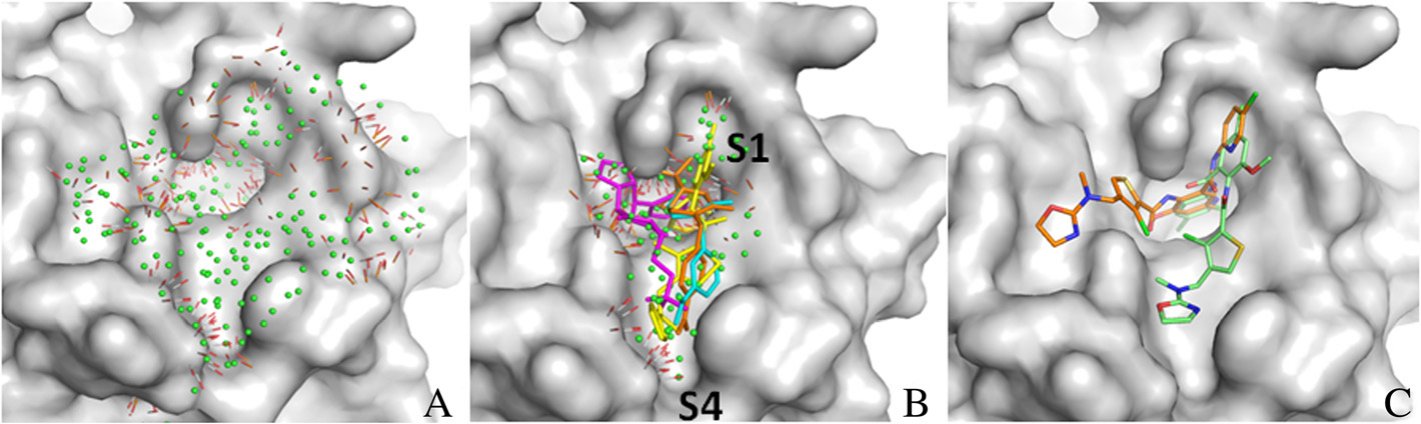
**Example of results obtained by confined docking protocol. (A)** All protein-based pharmacophores identified based on protein structure of factor Xa (PDB: 1MQ6) without inclusion of information about known active ligands. The protein surface is colored as grey. Protein-based pharmacophores are shown as lines for hydrogen bond donor/acceptor and small green spheres for hydrophobic elements. **(B)** A subset of the protein-based pharmacophores selected from **(A)** with the consideration of three known active ligands. The X-ray binding poses of known ligands are shown in stick representation. **(C)** Ligand binding poses predicted by PharmDock for co-crystallized ligand in PDB structure 1MQ6. Orange: Ligand binding pose predicted in the unbiased docking setting with all identified protein-based pharmacophores. Green: Ligand binding pose predicted with the selected pharmacophores showing excellent agreement (1.8 Å RMSD) with the X-ray pose (yellow ligand in **B**).

To demonstrate the potential of confined docking, we chose the inhibitor 3-chloro-N-[4-chloro-2-[[(5-chloro-2-pyridinyl)amino]carbonyl]-6-methoxyphenyl]-4-[[(4,5-dihydro-2-oxazolyl)methylamino]methyl]-2-thiophenecarboxamide complexed with human factor Xa (PDB-code: 1MQ6) as an example. Unbiased docking to all protein-based pharmacophores identified for factor Xa (fXa) (Figure 10A) resulted in the predicted docking pose which significantly deviates (RMSD = 7.7 Å) from the native pose. When several known factor Xa inhibitors were overlaid within the protein binding site, we found that they all bind within the groove formed by the S1 and S4 sub-pocket of fXa (Figure 10B). Based on this observation, only the pharmacophores within such groove were selected for confined docking simulation (Figure 10B). Using omega-generated conformers as input for confined docking the ligand of the 1MQ6 X-ray structure was accurately reproducing the native pose of this ligand with a 1.8 Å RMSD to the X-ray pose (Figure 10C).

**Figure 11:**
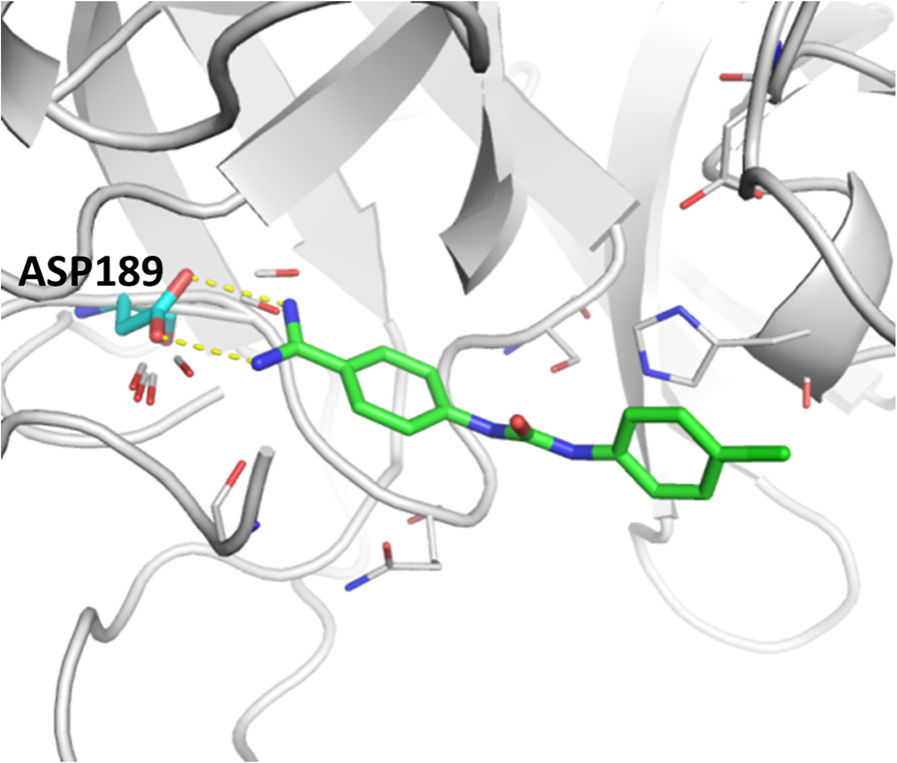
**Example of critical protein-ligand contact used as constraint in docking.** The aspartate residue 189 of the specificity pocket in the trypsin active site forms strong hydrogen bonds with the amidinophenyl moiety of trypsin inhibitors (PDBID: 1BJU). An example inhibitor co-crystallized with trypsin is shown in green. Yellow dashes represent the hydrogen bonds. Red-white lines represent the favorable locations (subset of protein-based pharmacophores) where a hydrogen bond donor groups from the ligands can interact with the hydrogen bond acceptor groups of the Asp residue.

In the virtual screening experiment, serine proteases turned out to be a difficult protein family for PharmDock with lower AUC values compared to the other docking programs (Figure 9). Among the serine proteases, trypsin only achieved an AUC value of 0.43. The aspartate residue (Asp189 in Figure 11) located in the active site of trypsin forms a specificity pocket that is responsible for attracting and stabilizing positively charged lysine or arginine side-chains in endogenous substrates. Non-covalent trypsin inhibitors with amidinophenyl moiety are also found to form stable hydrogen bonds with Asp189 [35,36] (Figure 11). Therefore, we hypothesized that a constraint docking simulation that requires interactions with residue Asp189 will lead to improved virtual screening results. To test this idea, we selected the six hydrogen bonding pharmacophores identified around the carboxylic acid group of Asp189. The selected pharmacophores represent the favorable locations for potential ligands to form hydrogen bonds with Asp189 (Figure 11). Re-running PharmDock using the constraint docking setting resulted in a significant increase in enrichment compared to the unbiased docking simulation, supported by an increase of the AUC from 0.43 for unbiased to 0.54 for constraint virtual screening.

## Conclusions

Starting from pre-generated ligand and protein-based pharmacophores, we extended our pharmacophore-based pose sampling and ranking into a docking program, named PharmDock. PharmDock’s performance in ligand binding pose prediction and binding energy estimation was tested using the PDBbind core set. We found that the presence of the native ligand conformation greatly influenced PharmDock’s performance in pose prediction: when native ligand conformation was provided together with the low energy conformations in docking, PharmDock was able to reproduce the native binding pose within an RMSD_top_ ≤ 2 Å for 56% of the protein-ligand complexes. This success rate dropped to 37% when the native ligand conformation is excluded from the docking inputs. Interestingly, the influence of the native ligand conformation was not that dramatic for binding energy estimation. Linear correlations between the predicted binding scores and the experimentally measured binding affinities were observed. The Pearson correlation coefficient of 0.580 and 0.567 were reached with and without the presence of native ligand conformations in the docking input.

We also measured PharmDock’s ability in ranking different ligands bound to the same protein according to their binding affinities. This test is directly related to the virtual screening study where the rankings rather than the absolute binding energies are critical to distinguish potentially active ligands from decoys. PharmDock was able to correctly rank the ligands bound to the same protein according to their binding affinities for 42% of the tested proteins. Subsequent test of PharmDock in virtual screening on 29 targets of DUD dataset yielded an average AUC of 0.61. Dependent on the size of protein binding site and ligand molecule, the required computation time needed to dock one ligand using PharmDock varies between 0.5 and 7 min for the DUD dataset on a single core of a 2.5 GHz Quad Core AMD2380 computer. The average time required for docking was about 2.7 min per ligand.

Many docking programs have been developed since the establishment of the computer-aided molecular design field. While we understand that due to many influencing factors a fair comparison between different docking programs is quite difficult [37], we chose to compare PharmDock with several widely used docking programs evaluated by other studies so we can better understand PharmDock’s weaknesses and strengths. Using a simple empirical scoring function, PLP, PharmDock’s performances in binding pose prediction and binding energy estimation were comparable to or better than many widely used docking programs. Whereas PharmDock provided a high Pearson correlation coefficient between the estimated binding energy and experimentally measured binding affinities compared to other programs, the overall binding affinity prediction is still not accurate enough for drug optimization purposes. This difficulty in predicting binding affinities is also reflected in its mediocre performances in ranking ligands bound to the same protein and of virtual screening experiments, although comparable to other available docking methods. A more sophisticated scoring function is still necessary for improving PharmDock’s performance for separating physically similar ligands with distinct biological affinity. Furthermore, the comparison between the Native-Seeded and Omega-Only results also indicates that a better sampling of the ligand conformations, in particular for large, flexible ligands would improve PharmDock’s performance for this class of compounds. Last but not least, it has also been shown that the inclusion of protein flexibility and dynamics using the “ligand model concept” recently developed in our group can significantly improve the enrichment of virtual screening experiment [38]. Future version of PharmDock will be combined with this “ligand model concept” to take protein flexibility and dynamics into consideration.

To make PharmDock accessible to any researcher in the field of biological and medicinal chemistry, we developed an open access PyMOl GUI for PharmDock. Both PharmDock and the PyMOl GUI can be downloaded from http://people.pharmacy.purdue.edu/~mlill/software/pharmdock. Two new features, confined docking and constraint docking, were built into the GUI. The idea is to provide users with the flexibility to include their expert knowledge about the target protein into the docking simulations. Two examples of these features demonstrated their usefulness in binding pose prediction and virtual screening.

## Availability and requirements

**Project name**: PharmDock

**Project home page**: http://people.pharmacy.purdue.edu/~mlill/software/pharmdock

**Operating system(s)**: Linux

**Programming language**: C, Python

**Other requirements**: OpenEye Omega (version 2.2.0 or newer), Python (version 2.4 or newer), Scipy, Numpy, OpenBabel (with Python bindings), PyMOL (tested on version 1.1r1 and 1.5.0)

**License**: GNU GPL v3

**Any restrictions to use by non-academics**: None

## Competing interests

The authors declare that they have no competing interests.

## Author’s contributions

BH developed PharmDock and PharmDock plugin, prepared the data sets, tested PharmDock, and drafted the manuscript. MAL participated in the development of PharmDock and PharmDock plugin, the discussion of the results, and helped to draft the manuscript. Both authors read and approved the final manuscript.
